# A Rare Aldosterone-Producing Adenoma Detected by ^68^Ga-pentixafor PET-CT: A Case Report and Literature Review

**DOI:** 10.3389/fendo.2019.00810

**Published:** 2019-11-29

**Authors:** Yunying Cui, Yushi Zhang, Jie Ding, Huiping Wang, Xiaoshen Ma, Ou Wang, Xiaoyan Chang, Hao Sun, Li Huo, Anli Tong

**Affiliations:** ^1^Key Laboratory of Endocrinology, Department of Endocrinology, National Health Commission of the People's Republic of China, Peking Union Medical College Hospital, Peking Union Medical College, Chinese Academy of Medical Sciences, Beijing, China; ^2^Department of Urology, Peking Union Medical College Hospital, Peking Union Medical College, Chinese Academy of Medical Sciences, Beijing, China; ^3^Department of Nuclear Medicine, Peking Union Medical College Hospital, Peking Union Medical College, Chinese Academy of Medical Sciences, Beijing, China; ^4^Department of Pathology, Peking Union Medical College Hospital, Peking Union Medical College, Chinese Academy of Medical Sciences, Beijing, China; ^5^Department of Radiology, Peking Union Medical College Hospital, Peking Union Medical College, Chinese Academy of Medical Sciences, Beijing, China

**Keywords:** aldosterone-producing adenomas, blood pressure, parathyroid hormone, rare area, ^68^Ga-pentixafor PET-CT

## Abstract

**Context:** Primary aldosteronism represents an important and common cause of hypertension and is characterized by autonomous aldosterone secretion that results in severe hypertension and hypokalemia. Nonetheless, its manifestations are atypical in some cases, which renders its diagnosis difficult.

**Case Description:** Presented in this report is a Chinese female patient with blood pressure in the high-normal range, and her parathyroid hormone was significantly elevated. Elevated plasma aldosterone concentration plus suppressed plasma rennin activity was suggestive of primary aldosteronism. ^68^Ga-pentixafor positron emission tomography/computed tomography revealed an aldosterone-producing adenoma, which was globally the second of its kind ever reported so far. Moreover, the tumor was located in an extremely rare area.

**Conclusions:** Patients with primary aldosteronism may present with normal or high-normal blood pressure and a significantly elevated parathyroid hormone. ^68^Ga-pentixafor PET/CT is potentially a helpful tool for the non-invasive characterization of patients with primary aldosteronism.

## Background

Primary aldosteronism (PA) represents an important and increasingly common cause of hypertension and is characterized by excessive secretion of aldosterone, which leads to hypertension, cardiovascular damage, sodium retention, suppressed plasma renin, and increased potassium excretion that may result in hypokalemia. PA is commonly caused by an aldosterone-producing adenoma (APA), unilateral or bilateral adrenal hyperplasia (BAH), or in rare cases, adrenal carcinoma or familial hyperaldosteronism. Over 90% of PA cases are attributable to either BAH or APA. The distinguishing between bilaterality and unilaterality of the condition has been diagnostically challenging and dictates the treatment choices. In PA patients, hypertension is usually much more frequent than hypokalemia. Only 50% of APA patients and 17% of BAH patients reportedly had hypokalemia. Normotensive PA was reported but was extremely rare (1).

Presented here is a Chinese female patient with extremely rare clinical findings, including severe hypokalemia with high-normal blood pressure according to the 2018 ESC/ESH Guidelines for the management of arterial hypertension (2), substantially elevated parathyroid hormone (PTH), and an APA at an extremely rare site. And the tumor was detected by ^68^Ga-pentixafor positron emission tomography/computed tomography (PET/CT). ^68^Ga-pentixafor, as a tracer, has been recently used for the diagnosis of APA.

## Case Presentation

A 28-year-old Chinese female was admitted to a local hospital with a history of muscular weakness for over 4 months. She was found to have persistent hypokalemia (the minimum serum potassium levels was 1.9 mmol/L with high-normal blood pressure (130/70 mmHg). She had been treated with potassium supplement (80 mmol daily), but not responded well to the treatment, with the serum potassium levels being lower than 3.0 mmol/L. She was referred to our hospital for further examination.

Physical examination showed a blood pressure of 120–140/70–90 mmHg. Her body mass index was 18.8 kg/m^2^. Routine laboratory tests revealed a low serum potassium level (3.0 mmol/L upon potassium supplement) with a relatively high urinary potassium excretion (110.4 mmol/24 h). Of note, her serum calcium levels were at the lowest limit of normal values, while PTH was significantly elevated. More biochemical findings are detailed in Table 1. Aldosterone-to-renin ratio was >30 ng/dl per ng/ml·h, and the plasma aldosterone concertration (PAC) was over 15 ng/dl, with a suppressed plasma renin activity (PRA) indicative of PA. She was further subjected to captopril test. After taking captopril, her PAC remained at a high level (declined only 8.7%) and PRA was still low (Table 1).

**Table 1:** Laboratory findings before and after surgery.

	**Before surgery**	**After surgery**	**Reference values**
Serum K(mmol/L)	2.7^a^	4.7	3.5–5.5
Serum Na (mmol/L)	140	136	135–145
Serum Cl (mmol/L)	100	102	96–111
Serum Ca (mmol/L)	2.02	2.50	2.13–2.70
SerumiCa(mmol/L)	1.06	1.18	1.10–1.20
Serum P(mmol/L)	1.2	1.2	1.1–1.3
Urinary K (mmol/24h)	110	–	–
Urinary Na (mmol/24h)	252	–	–
Urinary Ca(mmol/24h)	15.7	1.9	2.7–7.5
ALP(U/L)	72	60	35–100
β-CTX(ng/ml)	0.9	0.4	0.21–0.44
25-OHVitaminD(ng/ml)	5.7	10.2	8.0–50.0
PTH(pg/ml)	269	63	12–65
Upright PRA(ng/ml·h)	0.11	0.91	0.93–6.56
Upright PAC(ng/dl)	23.5	13.9	6.5–29.6
Captopril test			
Before treatment of captorpril			
PRA (ng/mL·h)	0.01	–	–
PAC (ng/dl)	22.1	–	–
After treatment of captorpril			
PRA (ng/ml·h)	0.01	–	–
PAC (ng/dl)	20.2	–	–
Cortisol (ug/dl)	15.7	15.6	4.0–22.3
ACTH(pg/ml)	35	72	0–46

a *Potassium supplement (55 mmol daily). K, Potassium; Na, Natrium; Cl, Chlorine; Ca, serum-bound calcium; iGa, isolated calcium; P, Phosphorus; ALP, Alkaline phosphatase;β-CTX, βcross-linked C-telopeptide of type I collagens; PTH, Parathyroid hormone; PRA, Plasma renin activity; PAC, Plasma aldosteroneconcertration; ACTH, Adrenocorticotropic hormone*.

**Figure 1:**
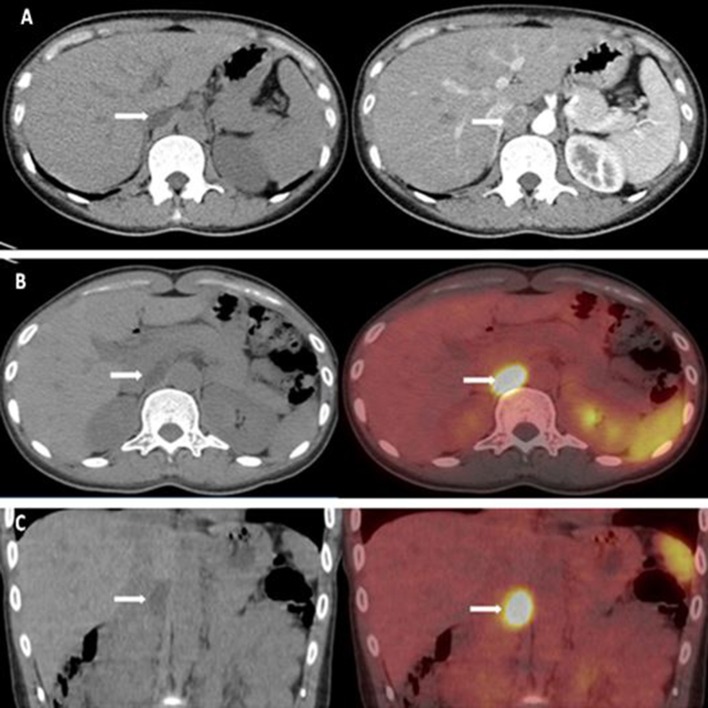
**(A)** CT scan revealed a 21 × 13 mm low-density mass (about 9 HU). The enhanced adrenal gland CT revealed a significant enhancement of the mass (about 70 HU). **(B,C)** ^68^Ga-pentixafor PET/CT revealed that the maximum standardized uptake was 22.83.

**Figure 2:**
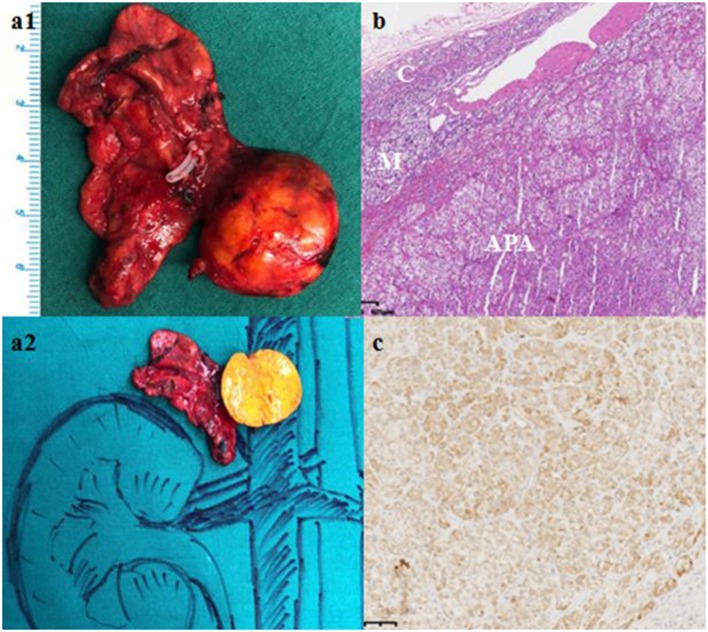
Gross pathological, histological and immunohistochemical findings. **(a)** Gross pathology revealed a 61 × 27 × 8 mm golden-yellow adenoma, which was connected to the right adrenal gland. **(b)** Hematoxylin-eosin staining, C: Normal adrenal cortex, M: Adrenal medulla; APA: Aldosterone-producing adenoma. **(c)** Immunohistochemical staining with CYP11B2.

Computed tomography (CT) scan revealed a 21 × 13 mm low-density mass (9 Hounsfield units), which was adjacent to the inner edge of the right adrenal junction, behind the inferior vena cava, and in front of the right diaphragmatic crus. The contrast-enhanced CT showed a tumor mass with significantly enhanced uptake (70 Hounsfield units). CT exhibited that the mass seemed to bear little relation with the right adrenal gland. The diagnosis of APA was not definitively established. Then, the patients underwent adrenal regional ^68^Ga-Pentixafor PET/CT imaging, which revealed a remarkably elevated uptake of the mass, with the maximum standardized uptake being 22.83, which strongly suggested an APA (Figure 1). Right adrenalectomy was performed upon obtaining consent from the patient. Pathological examination revealed a golden-yellow tumor with intact capsule sized 34 × 25 × 13 mm and connected to the right adrenal gland (Figure 2). Light microscopy showed a clearly-defined nodule in the adrenal cortex, with a fibrous tissue envelope. The nodule mainly contained two kinds of cells: clear cells and eosinophils. The cytoplasm was abundant, with no obvious atypia and pathological mitosis in the cells. The adrenal tissues surrounding the nodule were atrophic. Additionally, immunohistochemical staining with CYP11B2 yielded positive results, which strongly suggested a pathological diagnosis of APA (Figure 2). Genetic analysis showed that the patient had somatic mutation, i.e., *KCNJ5* p.G151R, a hotspot mutation. After the surgery, the patient's blood pressure ranged between 99-119/70 and 80 mmHg, and the serum potassium returned to normal without potassium supplementation. Other biochemical parameters, such as serum calcium, PTH, PAC and PRA also returned to normal at 3 months follow-up after surgery (Table 1). Informed consent was obtained from the patients and her family members for the publication of this case report.

## Discussion

In this report, we presented an APA patient with extremely rare clinical findings, which made its diagnosis difficult. The patient had severe hypokalemia with normotension or prehypertension. And her PTH level was significantly elevated. Moreover, the adenoma of the patient was located in an extremely rare area. We used ^68^Ga-pentixafor PET/CT and confirmed that the lesion was an APA. ^68^Ga-pentixafor, as a novel tracer, was first applied in 2018 for imaging the CXC chemokine receptor type 4 (CXCR4) that was highly expressed in aldosterone-producing cells.

Normotensive PA was initially described by Brooks et al. (3), and is still extremely rare. The exact rate of normotension in PA patients is unknown. Only Yuji *et al*. studied the prevalence of PA in prehypertensive patients, and revealed that, at least 6.8% of prehypertensive subjects (3 of 44 subjects) had PA (4). The ratio might be overestimated because the prevalence of PA increased with the severity of hypertension, from 3.9% in stage 1 hypertension to 11.8% in stage 3 hypertension. Médeau et al. compared 10 normotensive PA patients with 168 hypertensive PA patients in a retrospective study, and found that all of the 10 normotensive patients were women and all had an adrenal adenoma. Their body mass index was significantly lower, and mean tumor diameter was greater in the normotensive patients than in their hypertensive counterparts bearing an adenoma (5). Likewisely, this patient was a woman and had a low body mass index (18.8 kg/m^2^). A number of factors might contribute to normotension or prehypertension in PA patients. First, in PA patient, hyper-production of aldosterone causes sodium retention, thereby leading to hypertension. But in some patients, blood pressure remained normal because their spontaneous baseline levels were very low. Vantyghem et al. reported that the blood pressure of their normotensive patients significantly dropped after the surgery (6). With this patient, her blood pressure dropped conspicuously after surgery (from 120–140/70–90 mmHg to 99–119/70–80 mmHg). Another explanation is that they were at the early phase of the condition, and developed hypertension during later phases.

In 1985, Resnick et al. found, for the first time, that secondary hyperparathyroidism might be a clinical feature of PA (7). Subsequently, multiple studies observed that 24-h urinary calcium excretion was significantly higher in PA patients than in those with primary hypertension. This elevated calcium excretion led to lower serum calcium and then increased PTH level in PA patients. These abnormalities could be remedied by medications or surgery. In these reports, PTH elevation might vary but was marginal, with the mean PTH concentration ranging from 46 to 118 pg/ml (8, 9). The PTH concentration of our patient was 269 pg/ml, which was significantly higher than previously reported. Vitamin D deficiency in our patient might be responsible for the aggravation of her secondary hyperparathyroidism. Rossi et al. found that PTH elevation could help distinguish between BAH and APA, since the Youden Index of PTH was 80 ng/L, which corresponded to a sensitivity of 74% and a specificity of 82%, respectively (10). However, Jiang et al. reported that PTH level was not linked to the subtypes of PA in their study that enrolled 242 PA patients (9). The elevated PTH in PA patients might be ascribed to several factors. First, in PA patients, hypokalemia leads to metabolic alkalosis, and, as a result, the serum-bound calcium increased, which lowers the level of isolated calcium, and then raises PTH level. Second, in patients with PA, aldosterone induces Na^+^-Ca^2+^ exchange on renal tubules, which increases urinary calcium excretion and decreases serum calcium levels. Third, mineralocorticoid receptor was detected in human parathyroid tissue. And aldosterone can directly stimulate the production of PTH. Meanwhile, PTH can reciprocally regulate aldosterone secretion. PTHR-1 was found to express at both mRNA and protein levels in APA tissues. And PTH could enhance the secretion of aldosterone in human adrenocortical cells (11).

In this case, adenoma was located at an extremely rare site.^68^Ga-pentixafor PET-CT revealed a mass, which, with its clinical features, strongly suggested APA. Ectopic aldosteronoma was considered at first because of the location of the mass, as shown by CT scan. Postoperatively, we dropped the diagnosis since the adenoma was pathologically found to be connected to the right adrenal gland.

Surgical treatment of the condition asks for differentiation of APA from BAH, which is difficult to achieve with CT alone. This is especially true of micro-APAs, which either go undetected by CT or might be mis-diagnosed as BAH. As a result, the patient might be excluded from appropriate surgery. In contrast, CT could not provide information on the functional status of unilateral adrenal masses (1), and might incorrectly suggest adrenalectomy for patients with BAH complicated with a non-functional adenoma. The prevalence of adrenal neoplasms/incidentaloma stands somewhere between 1 and 8.7% (12), depending on the age of patients, and most adrenal masses are non-functional. Adrenal vein sampling remains the standard procedure for subtype classification of PA, despite its high cost and limited availability only in some clinical centers. Moreover, adrenal vein sampling is an invasive technique that is not well standardized among centers and heavily depends on the expertise of the interventional radiologist.

Alternative strategies for lateralization, including nuclear medicine techniques, are being developed and introduced into clinical practice (Table 2). Scintigraphy with ^131^I-iodomethyl-norcholesterol (NP-59) was traditionally used for imaging of Conn's adenomas. However, it is no longer widely employed since it requires pretreatment with dexamethasone for up to 1 week to suppress the uptake of tracer by normal adrenal, and its resolution fails to image lesions <1.5–2 cm (17). ^11^C-metomidate, a potent inhibitor of 11-hydroxylase and aldosterone synthase, has been introduced as a PET radiotracer and is considered promising. However, ^11^C-metomidate has only a half-life of 20 min, which substantially limits its clinical application. In 2018, Heinze et al. observed, for the first time, that CXCR4 was highly expressed in aldosterone-producing cells in normal adrenals and APA tissues, its expression was strongly correlated with the expression of CYP11B2 (aldosterone synthase), while CXCR4 expression was not detected in most of the non-functional adrenal adenomas. The specific CXCR4 ligand ^68^Ga-pentixafor has recently been introduced as radiotracer for molecular imaging of CXCR4 expression and showed strong and specific binding to APAs. They further investigated 9 APA patients by employing ^68^Ga-pentixafor PET/CT. The tracer uptake was found to be significantly higher in patients with APAs than in those with cortisol-producing adenomas and non-functional adenomas (16). We utilized ^68^Ga-pentixafor PET/CT to detect APA in this patient. This was the second case in which the novel approach was used for APA diagnosis. ^68^Ga-pentixafor PET/CT may be a highly promising tool for non-invasive characterization of patients with APAs and warrants more research to verify its value.

**Table 2:** Comparison of various adrenal images in the diagnosis of primary aldosteronism.

	**Theory**	**Cases**	**Sensitivity**	**Specificity**	**Limitations**	**References**
High-resolution CT	–	–	~80–85%	~70–75%	Limited sensitivity for microadenomas Provideno information on the functionality	(13)
NP-59	Cholesterol as material for the synthesis of steroid hormones	686	86%	78%	Tedious examination process Limited sensitivity for microadenomas	(14)
^11^C-metomidate PET/CT	the inhibitor of CYP11B1 and CYP11B2	39	76%	87%	Half-life (20 min)	(15)
^68^Ga-pentixafor PET/CT	CXCR4 express in APA	9	89%	85%	-	(16)

*NP-59, ^131^I-6β-iodomethyl-19-norcholesterol; PET/CT, positron emission tomography/computed tomography; CYP11B1, 11b-hydroxylase; CYP11B2, aldosterone synthase; CXCR4, CXC chemokine receptor type 4; APA, aldosterone-producing adenomas*.

In conclusion, we presented a case of APA with some extremely rare clinical features, including severe hypokalemia with high-normal blood pressure, substantially elevated serum PTH, and an APA mass located in an extremely rare site. Positive ^68^Ga-pentixafor PET/CT finding was very helpful for the diagnosis of APA.

## Data Availability Statement

The raw data supporting the conclusions of this manuscript will be made available by the authors, without undue reservation, to any qualified researcher.

## Ethics Statement

Written informed consent was obtained for the publication of any potentially identifiable images or data included in this article.

## Author Contributions

YC and YZ performed the study and drafted the manuscript. AT and LH contributed to the concept and design for the study. HW, XM, and OW contributed to the manuscript preparation. HS and JD prepared Imaging results. XC prepared histopathological results. All authors contributed to critical interpretation of data and the final draft of the manuscript.

### Conflict of Interest

The authors declare that the research was conducted in the absence of any commercial or financial relationships that could be construed as a potential conflict of interest.
